# Pressures in Archaeal Protein Coding Genes: A Comparative Study

**DOI:** 10.1002/cfg.246

**Published:** 2003-02

**Authors:** Sujay Chattopadhyay, Satyabrata Sahoo, William A. Kanner, Jayprokas Chakrabarti

**Affiliations:** 1 Department of Theoretical Physics Indian Association for the Cultivation of Science Jadavpur Calcutta 700 032 India; 2 Atlanta VA Medical Center and Emory University School of Medicine 1670 Clairmont Road (111B) Decatur GA 30033 USA

## Abstract

Our studies on the bases of codons from 11 completely sequenced archaeal genomes show that, as we move from GC-rich to AT-rich protein-coding gene-containing species, the differences between G and C and between A and T, the purine load (AG content), and also the overall persistence (i.e. the tendency of a base to be followed by the same base) within codons, all increase almost simultaneously, although the extent of increase is different over the three positions within codons. These findings suggest that the deviations from the second parity rule (through the increasing differences between complementary base contents) and the increasing purine load hinder the chance of formation of the intra-strand Watson–Crick base-paired secondary structures in mRNAs (synonymous with the protein-coding genes we dealt with), thereby increasing the translational efficiency. We hypothesize that the ATrich protein-coding gene-containing archaeal species might have better translational efficiency than their GC-rich counterparts.


**Comparative and Functional Genomics**
*Comp Funct Genom* 2003; **4**: 56–65.
Published online in Wiley InterScience (www.interscience.wiley.com). **DOI:** 10.1002/cfg.246

**Conference Paper**

### Pressures in archaeal protein coding genes: a comparative study

Sujay Chattopadhyay[1]*, Satyabrata Sahoo[1], William A. Kanner[2] and Jayprokas Chakrabarti[1]

[1] *Department of Theoretical Physics, Indian Association for the Cultivation of Science, Jadavpur, Calcutta 700 032, India*
[2] *Atlanta VA Medical Center and Emory University School of Medicine, 1670 Clairmont Road (111B), Decatur, GA 30033, USA*

**Correspondence to:*
*Sujay Chattopadhyay, Department of Theoretical Physics, Indian Association for the Cultivation of Science, Jadavpur, Calcutta 700 032, India.*
*E-mail: tpsc@mahendra.iacs.res.in*

### Abstract

**Our studies on the bases of codons from 11 completely sequenced archaeal genomes show that, as we move from GC-rich to AT-rich protein-coding gene-containing species, the differences between G and C and between A and T, the purine load (AG content), and also the overall persistence (i.e. the tendency of a base to be followed by the same base) within codons, all increase almost simultaneously, although the extent of increase is different over the three positions within codons. These findings suggest that the deviations from the second parity rule (through the increasing differences between complementary base contents) and the increasing purine load hinder the chance of formation of the intra-strand Watson–Crick base-paired secondary structures in mRNAs (synonymous with the protein-coding genes we dealt with), thereby increasing the translational efficiency. We hypothesize that the AT-rich protein-coding gene-containing archaeal species might have better translational efficiency than their GC-rich counterparts. Copyright © 2003 John Wiley & Sons, Ltd.**

Received: 13 September 2002
Accepted: 25 November 2002

**Keywords: archaeal protein coding genes; GC and AT contents; second parity rule; purine load; persistence**

### Introduction

Recent knowledge of the complete sequences of some archaeal genomes has led us to a comparative study of different sequence features among archaeal species. Archaea do share similarities with bacteria, viz., large circular genomes, sometimes with some circular plasmids and the absence of nucleosomal structure, a single initiation site for genome replication (Myllykallio *et al*., 2000), etc. However, the archaeal species also show closeness to eukaryotes. There are similarities in DNA and RNA polymerases, in ribosomal RNA and proteins, in several other proteins related to information processes, and in the presence of TATA box binding sites etc. (Pühler *et al*., 1989; Zillig *et al*., 1993; Brown and Doolittle, 1997).

The separate taxonomic status of archaea (Woese and Fox, 1977; Woese, 1987; Woese *et al*., 1990; Olsen and Woese, 1993; Olsen *et al*., 1994) owes a lot to some unique features, such as the presence of prenyl ether lipids instead of acyl ester lipids, and of a tiny large subunit ribosomal protein LX, the absence of HSP90 chaperone, and the presence of a split in RNA polymerase A (Cavalier-Smith, 2002). Considering the similarities and the differences, it is believed that archaea narrow the gap between bacteria and eukaryotes (Keeling and Doolittle, 1995; Olsen and Woese, 1997; Gaasterland, 1999).

The 11 completely sequenced archaeal genomes — both euryarchaeotes and crenarchaeotes– were the subject of our study. The euryarchaeote species were *Halobacterium* sp. NRC-1 (*Halo*), *Archaeoglobus fulgidus* (*Aful*), *Methanobacterium thermoautotrophicum* $\Delta$H (*Mthe*), *Methanococcus jannaschii* (*Mjan*), *Thermoplasma acidophilum* (*Taci*), *Thermoplasma volcanium* GSS1 (*Tvol*), *Pyrococcus abyssi* (*Paby*) and *Pyrococcus horikoshii* OT3 (*Phor*); while the crenarchaeote species

Copyright © 2003 John Wiley & Sons, Ltd.

were *Aeropyrum pernix* K1 (*Aper*), *Sulfolobus solfataricus* (*Ssol*) and *Sulfolobus tokodaii* (*Stok*). The short names in parentheses have been used in the rest of this paper. For each genome we analysed a single large DNA sequence obtained by concatenating all of the protein coding genes. During concatenation, when we found 'complementary' regions on the GenBank strand, we converted them into the protein-coding genes sitting on the other strand in the 5′ to 3′ direction, and considered those genes along with the protein-coding genes on the GenBank strand. Therefore, we concatenated all the protein-coding sequences from both strands, maintaining the coding direction. Our samples were these 11 concatenated sequences.

The main aim of this paper has been to isolate some of the pressures on these archaeal genes. Violations of the second parity rule, PR2 (Sueoka, 1995, 1999), were observed by applying PR2 to the single strand of DNA sequences. Here we investigated PR2 on the concatenated sequences of genes to study the global violations of PR2 within all the protein-coding genes from both strands taken together. Both G — C (the difference between G and C contents) and A — T (the difference between A and T contents) did get bigger with increasing AT content. We have shown, however, that there were other simultaneous pressures that, in effect, gave rise to an increase in purine load. This rise in purine load was not uniform over the codon sites. It was a spatially differentiated rise, with the load rising maximally on the 1st codon position, followed by the rise on the third site; while the interspecific variation in purine load was the least for the middle position of the codons. Accompanying these changes in base composition was the trend towards increasing persistence within codons with AT richness. While the presence of persistence was consistent with Chargaff's (1963) clustering rule, which suggested the clustering of individual bases to an extent larger than random expectations, we found that the major contributions to this clustering were through persistence between the first and third and between the second and third positions within codons. The persistence between the first and second did not contribute significantly towards this clustering. We showed the clustering in archaea was again a spatially differentiated process that did not occur uniformly over all the codon positions.

### Methods

The GenBank Accession Nos for the complete genome sequences of the archaeal species studied here are: AE004437, *Halo* (Ng *et al.*, 2000); AE000782, *Aful* (Klenk *et al.*, 1997); AE000666, *Mthe* (Smith *et al.*, 1997); L77117, *Mjan* (Bult *et al.*, 1996); AL139299, *Taci* (Ruepp *et al.*, 2000); BA000011, *Tvol* (Kawashima *et al.*, 1999); AL096836, *Paby* (Heilig R, 1999, unpublished, *Pyrococcus abyssi* genome sequence: insights into archaeal chromosome structure and evolution); BA000001, *Phor* (Kawarabayasi *et al.*, 1998); BA000002, *Aper* (Kawarabayasi *et al.*, 1999); AE006641, *Ssol* (She *et al.*, 2001); and BA000023, *Stok* (Kawarabayasi *et al.*, 2001).

### Usage of bases within codons

We studied the frequencies of occurrence of A, C, G and T in each of the three positions in codons. We also calculated the overall average frequencies of the four bases in all three positions. In each case, we measured the purine load, in terms of AG content, as well as the differences between A and T contents, and between G and C contents.

### Measuring similarity index

We studied the frequencies of occurrence of any nearest-neighbour codon-pairs. On this basis for each species we developed a 64 × 64 matrix. These matrices carried the footprints of nearest-neighbour selectional influence on codon usage. We measured the extent of similarity among them. If $C_{ij}(M1)$ and $C_{ij}(M2)$ were the values of any particular cell $C_{ij}$ (where both $i$ and $j$ run from 1 to 64) of matrices $M1$ and $M2$, respectively, the similarity index ($SI$) was given by:

$$SI = \left(\sum_{i=1}^{64}\sum_{j=1}^{64}[C_{ij}(M1) - C_{ij}(M2)]^2\right) \times \frac{100}{\sum C_{ij}} \quad (1)$$

where $\sum C_{ij}$ denoted the total number of cells in the 64 × 64 matrix.

### Measuring persistence index

Persistence meant a base did tend to be followed by the same base. We looked for the level of persistence within codons. Therefore, our definition

Copyright © 2003 John Wiley & Sons, Ltd. 

suggested that AAA, CCC, GGG and TTT are the most persistent codons; while codons such as ACA, TTG, and TCC, where two of the three bases were identical, did bring a somewhat lower level of persistence in the sequence. On the contrary, GCA, CAT, etc. were antipersistent codons (Chattopadhyay *et al.*, 2002). We computed the square of the number of any particular base within each codon along the sequence and the averaged value gave the persistence index (*PI*) within codons for that particular base. *PI*, therefore, was given by:

$$(PI)_b = \langle n_b^2(3) \rangle \quad (2)$$

where $b$ was any base (A, C, G, T), and $\langle n_b^2(3) \rangle$ could be $\langle n_A^2(3) \rangle$ or $\langle n_C^2(3) \rangle$ or $\langle n_G^2(3) \rangle$ or $\langle n_T^2(3) \rangle$. The '(3)' is to highlight the base length of codons, which is three.

### Results and discussion

### Base usage within codons

*Halo* was the most GC-rich protein-coding gene-containing species, followed by *Aper*, while both *Aful* and *Meth* have about 50% GC content. The remaining seven species were AT-rich; with *Ssol, Stok* and *Mjan* having maximum AT content. It might be worth noting here that GC richness in protein-coding genes did not correlate with increasing thermophilicity of species; e.g. the only mesophilic species dealt with here, *Halo*, was the most GC-rich gene-containing species. As the 11 species were placed in decreasing order of GC content separately for the three positions of codons, the extent of decrease was noted to be most pronounced for the third position of codons (Figure 1). It is well known that the third position of codons is the most susceptible to change over time. In fact, the codon usage study showed that with the increase in AT richness within genes, the archaeal species opted for increased usage of comparatively AT-rich synonymous codons, mostly differing in the third position of codons (e.g. for glycine the use of GGC and GGG were gradually overshadowed by GGA and GGT in AT-rich gene-containing species).

The averages of individual base content over the three codon positions showed that for all the 11 samples, %A exceeded %T; similarly %G was greater than %C except for *Halo* (Figure 1d). It was known from earlier studies of bacteria and primates that %A did exceed %T in protein-coding sequences (Mrázek and Kypr, 1994; Bell and Forsdyke, 1999). The archaeal samples were no exception in this regard. Let A − T be denoted by ΔW, and G − C by ΔS. In Table 1, the %(A + T) content averaged over all the three positions within codons is shown for 11 archaeal species. As we plotted ΔW and ΔS with increasing %(A + T) content for the 11 species, we found both the overall ΔW and the overall ΔS increased (Figure 2). Again, for samples with %(A + T) > %(G + C), ΔS > ΔW. For these AT-rich samples, ΔW stayed roughly the same. Taken together, we saw that C content reduced faster compared to G. Since ΔW increased with decreasing G + C, we conclude that A + G, i.e. the purine load, increased with increasing %(A + T) (Figure 3).

Interestingly, as we plotted the values of ΔW, ΔS and %(A + G) for first, second and third positions within codons and for all three positions taken together (Figures 2 and 3), we found the first position to be the strongest so far as the PR2 violations and purine load in protein coding genes were concerned. For the second position, both ΔW and ΔS values were negative in most cases, and species-wise purine load was minimum, with little interspecific variation except for *Mjan*. The third position showed some negative ΔW and ΔS values and weak purine load compared to the first position, but the increase in purine load from *Halo* (42.677%) to *Mjan* (55.447%) was extremely conspicuous. The first position always had high ΔW and ΔS values with considerable

**Table 1.** The %(A + T) content in the protein coding genes of 11 archaeal species

| Species | %(*A* + *T*) |
|---|---|
| Halo | 31.508 |
| *Aper* | 42.483 |
| *Mthe* | 49.44 |
| *Aful* | 50.643 |
| *Taci* | 52.715 |
| *Paby* | 54.844 |
| *Phor* | 57.683 |
| *Tvol* | 59.013 |
| *Ssol* | 63.514 |
| *Stok* | 66.416 |
| *Mjan* | 68.065 |

Copyright © 2003 John Wiley & Sons, Ltd. 

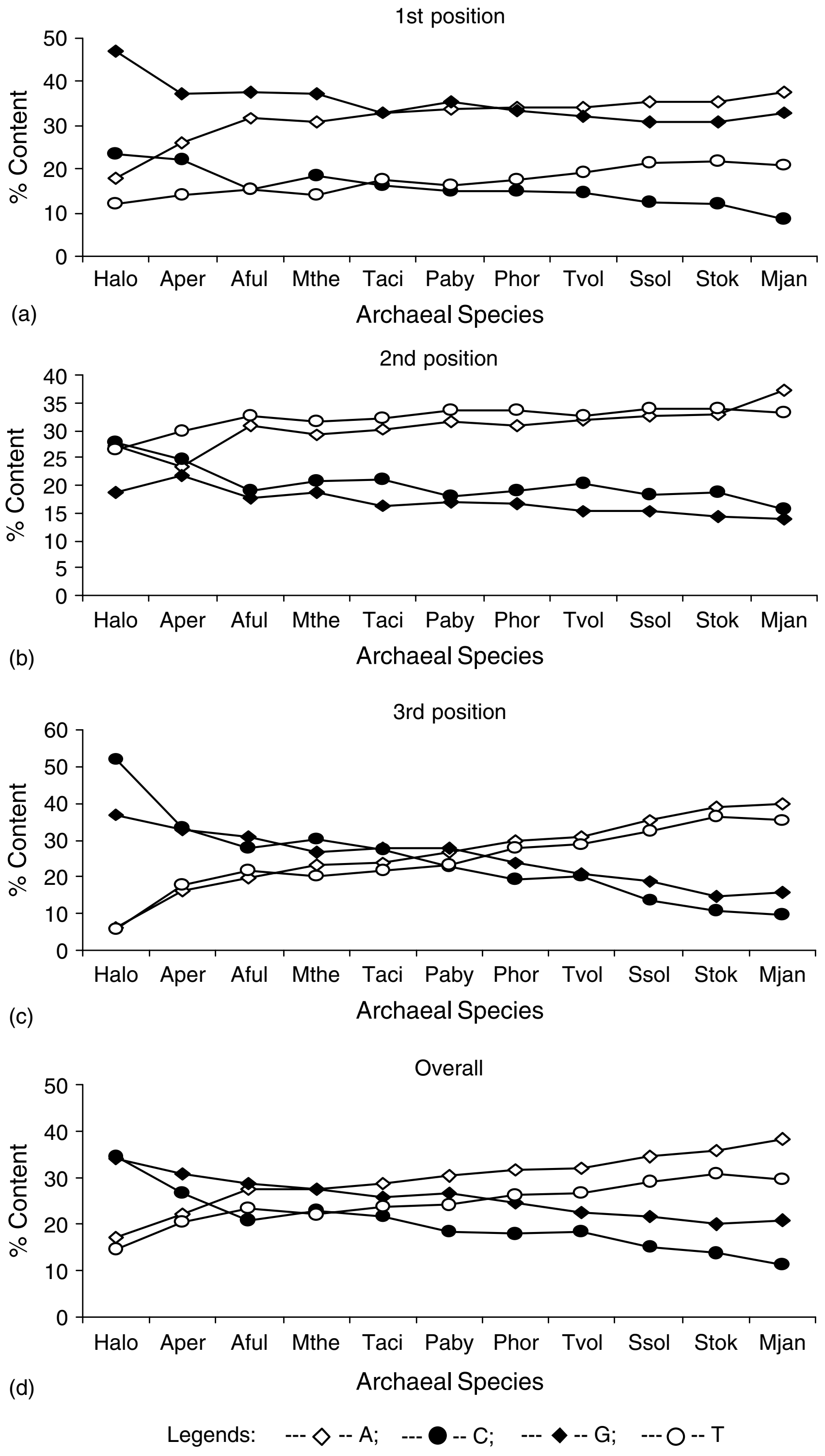

**Figure 1.** The base usage within codons for the archaeal species, separately for the first position (a), second position (b), third position (c) and also the overall average of all three positions (d)

Copyright © 2003 John Wiley & Sons, Ltd. 

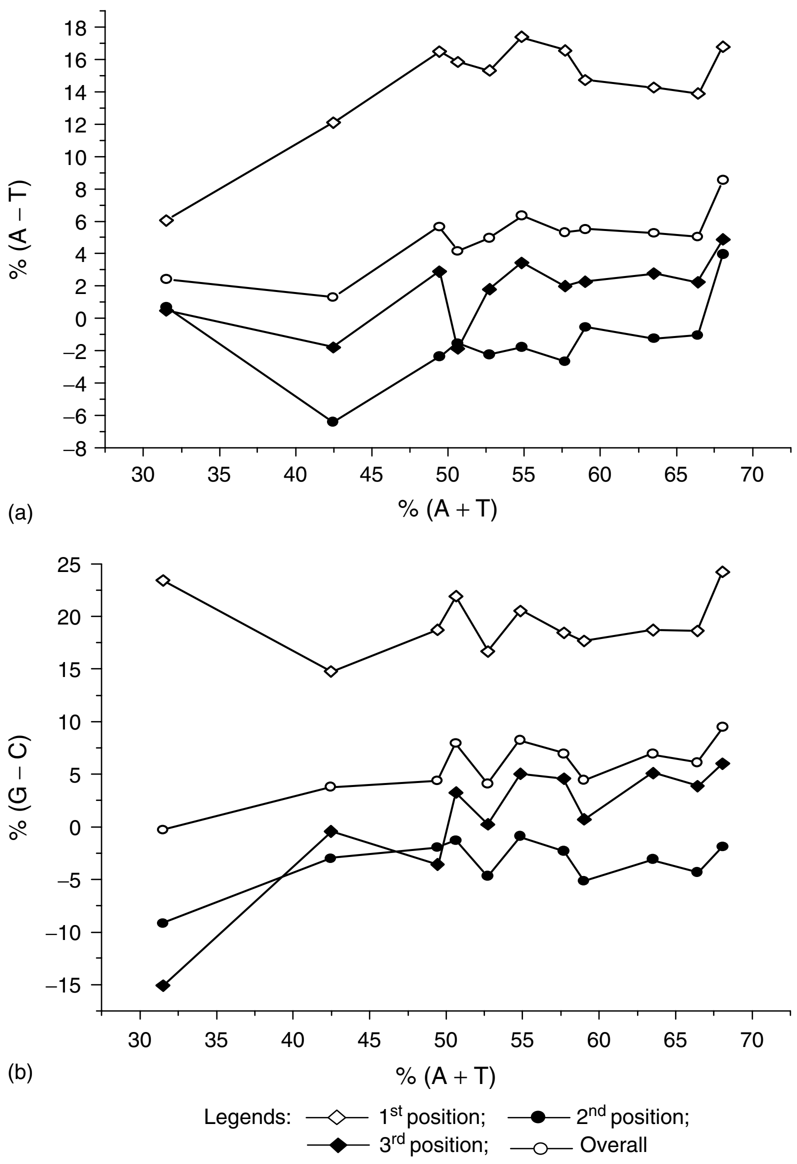

**Figure 2.** The PR2 violations as averaged over all the three positions within codons, and also in the first, second and third positions separately, are plotted with increasing average %(A + T) as in Table 1; (a) shows the ΔW, i.e. %(A − T) values, while (b) shows the ΔS, i.e. %(G − C) values purine load, which increased from ~63% to ~71% as %(G + C) decreased from *Halo* to *Mjan*.

### Comparison based on codon relatives

A considerable amount of the literature witnesses the regularity of context sensitivity in the usage of codons from different prokaryotes and eukaryotes (Irwin, Heck and Hatfield, 1995; Karlin and Mrázek, 1996; Berg and Silva, 1997; Antezana and Kreitman, 1999; McVean and Hurst, 2000; Fedorov *et al.*, 2002). We compared 64 × 64 matrices for any pair of species based on nearest-neighbour codon-pair frequencies to get SI for those two species. When simply viewed, we noted that the distance of 10 other species from *Halo*, based on *SI*, increased with AT richness; therefore, the *SI* between *Mjan* (the most AT-rich one) and *Halo* (the most GC-rich one) was found to be minimal (Figure 4).

Copyright © 2003 John Wiley & Sons, Ltd.

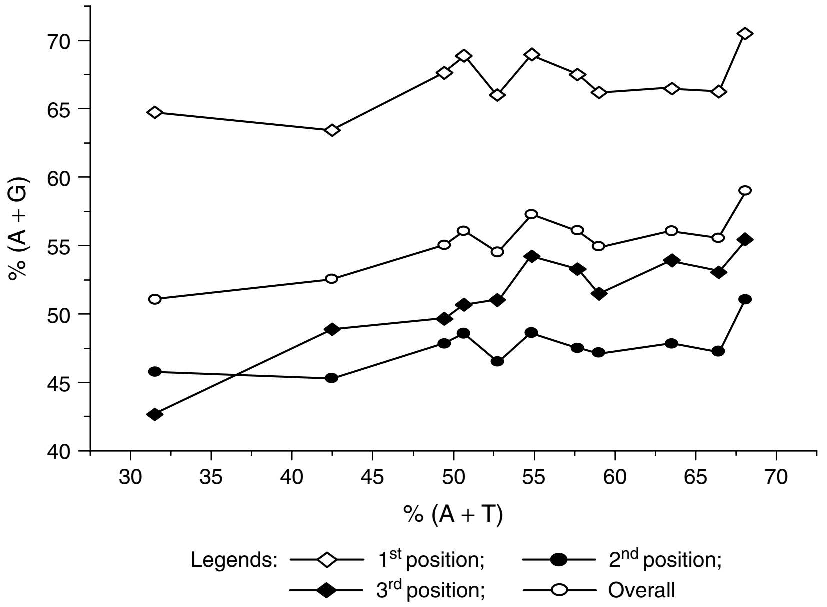

**Figure 3.** The purine load, i.e. %(A + G), as averaged over all the three positions within codons, and also in the first, second and third positions separately, are plotted with average increasing %(A + T), as in Table 1

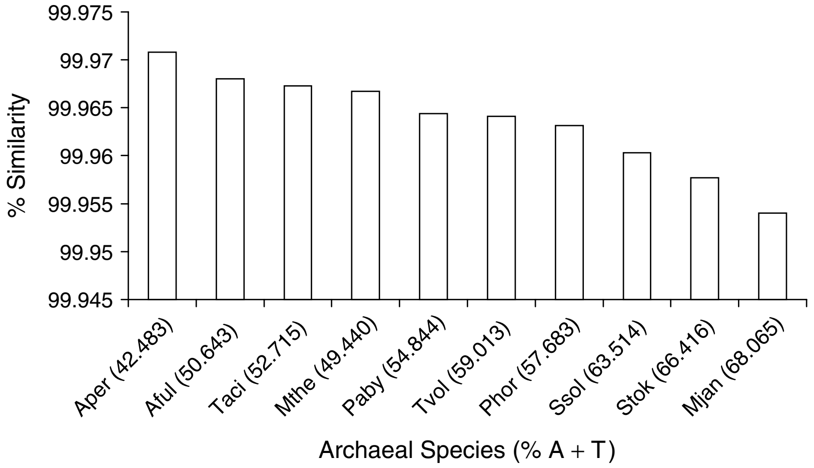

**Figure 4.** The *SI* values between *Halo* and each of the rest 10 species are shown. The bars are arranged along the *x*-axis in a decreasing order of the extent of similarity of the 10 species with *Halo*. The values within parentheses show the average %(A + T) for respective species, as in Table 1

### Persistence within codons

As expected, moving from GC-rich codon-containing species to AT-rich ones showed an increase in persistence for A and T channels, while antipersistence increased in G and C channels (Figure 5a). Interestingly, the rates of increase in persistence and antipersistence were not equal; and the former dominated. Therefore, the average over the sum of individual *PI* values, termed:

$$(PI)_{all}, \text{i.e } 1/4\sum_{b}\langle n_b^2(3)\rangle$$

showed an overall increase in persistence towards AT-rich codon-containing species (Figure 5b). The $(PI)_{all}$ showed somewhat lower values for *Taci*,

Copyright © 2003 John Wiley & Sons, Ltd.

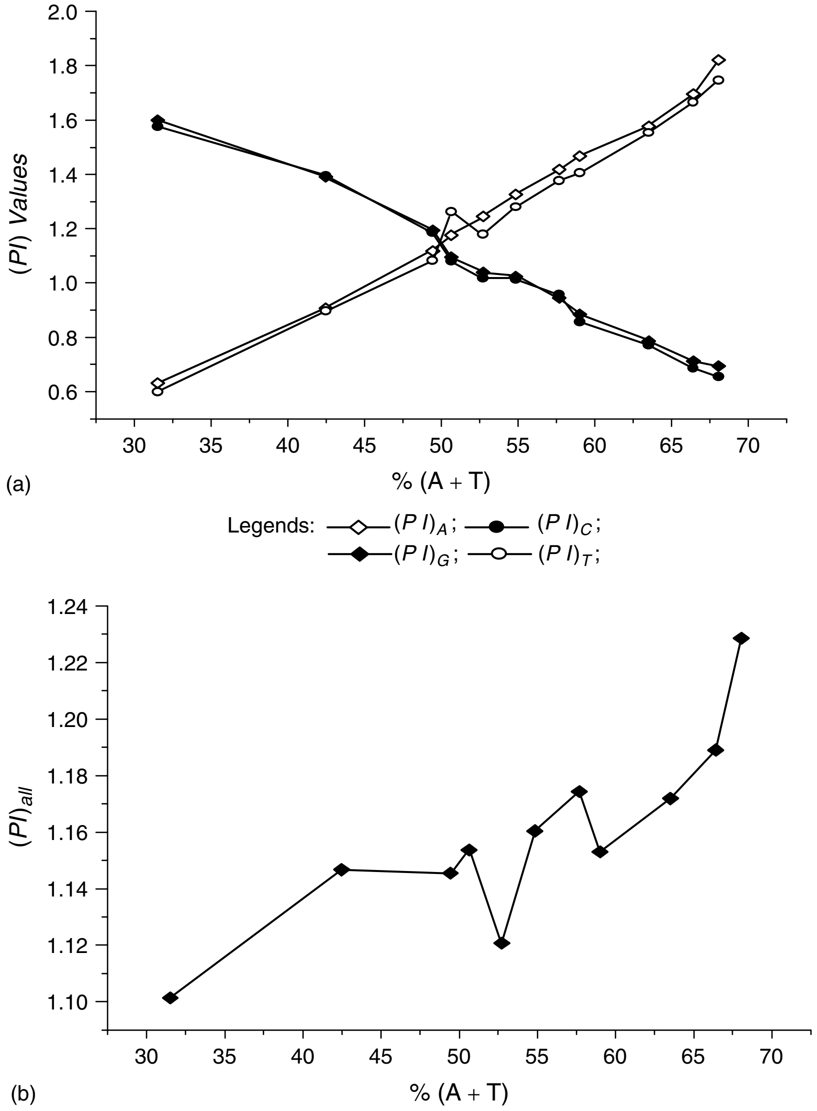

**Figure 5.** The *PI* values within codons are plotted with increasing average %(A + T), as in Table 1; (a) shows individual base persistence within codons and (b) shows the overall base persistence within codons (averaged over the sum of all four individual *PI* values). The notations used are described in the text placing it close to *Halo* based on the overall level of persistence within codons.

We thereafter traced which pair of bases within codons did hold maximum responsibility in imparting the trend in overall persistence. The study showed that paired bases 1 and 3, as well as 2 and 3, shared major and more or less equal contributions in increasing persistence within codons; while 1 and 2 contributed the least (Table 2). This suggested that there was a spatially differentiated 1-periodic (only due to the second and third positions) and a 2-periodic (due to the first and third positions) structure within codons. But, as we calculated the 1- and the 2-periodicities over the entire sequences, we found that the 1-periodicity values showed a prominent increasing trend, unlike 2-periodicity values, with decreasing GC content and increasing purine load (results not shown). Again, the extent of persistence between the third position of the previous codon and the first position of the next codon was really low, even lower than that between the first and second positions

**Table 2.** Periodicities as measured from the dinucleotide second moments. (*1–2*), (*2–3*), (*1–3*) denote the persistence between the first and second, the second and third, and the first and third positions within codons. (*3–1*) denotes the persistence between the third position of previous codon and the first position of the next codon

| | **(*1–2*)** | **(*2–3*)** | **(*1–3*)** | **(*3–1*)** |
|---|---|---|---|---|
| *Halo* | 2.40018 | 2.49966 | 2.50009 | 2.30088 |
| *Aper* | 2.40076 | 2.50001 | 2.5001 | 2.30129 |
| *Aful* | 2.50021 | 2.40079 | 2.89749 | 2.40065 |
| *Mthe* | 2.40137 | 2.50067 | 2.49994 | 2.30204 |
| *Taci* | 2.40086 | 2.50008 | 2.50006 | 2.30174 |
| *Tvol* | 2.40125 | 2.50052 | 2.50011 | 2.30236 |
| *Paby* | 2.40132 | 2.50048 | 2.49987 | 2.30211 |
| *Phor* | 2.40138 | 2.50064 | 2.50005 | 2.30216 |
| *Ssol* | 2.40075 | 2.50034 | 2.50024 | 2.30137 |
| *Stok* | 2.40092 | 2.50044 | 2.5003 | 2.30138 |
| *Mjan* | 2.40149 | 2.50073 | 2.49994 | 2.30292 |

within codons (Table 2). This implied the dominance of the intra-codon 1-periodicities over the inter-codon 1-periodicity. We noted that the values for *Aful* in Table 2 were distinctly different from other archaeal species: while the level of persistence between the second and third positions was much smaller than that in any other species, the persistence levels between all other pairs of positions were relatively higher than those for any other species.

### Hints for translational efficiency

The $\Delta$W and $\Delta$S values in Figure 2 led us to assess Szybalsky *et al.*'s (1966) transcription-direction rule and purine loading. The mRNA synonymous DNA sequences, precisely the protein-coding sequences that we dealt with here, were purine-rich (Dang *et al.*, 1998). This purine richness in mRNA reduces the formation of double-stranded RNA secondary structure (more precisely, the formation of intra-strand Watson–Crick base pairing). This presumably increases the corresponding translational efficiency (Lao and Forsdyke, 2000). The pressure in the 11 archaeal genomes, as observed here, resulted in increase of $\Delta$W and $\Delta$S with increasing AT content. Thus, the deviations from PR2 increased. These increases in PR2 deviations were important, but not sufficient. When we added the effects of PR2 together with $\Delta C > \Delta G$ with decreasing $G + C$, and $\Delta$W increasing with decreasing $G + C$, we arrived at the increase in purine load. Overall this pressure of increasing purine load in archaea reduces the strength of the mRNA secondary structure in comparatively AT-rich species; the strongest footprints being in the third position of codons where the positive correlation between the increase in AT content and the increase in purine load was found to be most conspicuous.

There was another important component to the pressures in archaeal protein coding genes, which related to persistence of bases within codons. This persistence, or clustering, was between the first and the third position and between the second and the third (Table 2). The relative weights of these two possibilities were roughly equal, both being greater than the one between the first and the second. The 'cluster rule' of Chargaff (1963) that the individual bases tend to cluster more than expected on a random basis is true, but curiously not between the first and the second position in the case of archaeal protein coding genes; furthermore, the level of persistence was even worse if we considered the third position of the previous codon and the first position of the next codon, suggesting the dominance of intra-codon 1-periodic persistence over inter-codon 1-periodic persistence.

The overall increase of $A + T$ at the expense of $G + C$ obviously increased the melting flexibility (Ussery, 2001), but it had consequences for mRNA secondary structure as well. Its importance was emphasized earlier in that a rigid secondary structure implied a limited coding potential (Salser, 1970; Ball, 1972, 1973). It was also noteworthy that while GC content of rRNA had a positive correlation with the optimum growth temperatures of thermophilic microbes (Dalgaard and Garrett, 1993; Forterre and Elie, 1993; Galtier and Lobry, 1997), the same sort of correlation was not found between their mRNA GC content and their optimum growth temperatures (Galtier and Lobry, 1997; Filipski, 1990). Bernardi and Bernardi (1986) suggested that genomic GC might have important roles in more fundamental adaptive processes and temperature remained unable to dictate the GC content. On the other hand, the genomic DNA might achieve its high thermal stability through its association with polyamines (Oshima *et al.*, 1990) or through relaxation of supercoiling (Friedman *et al.*, 1995). The increase in AT, in this view, might lead to greater

Copyright © 2003 John Wiley & Sons, Ltd. 

translational efficiency. This is in addition to what we have discussed earlier about purine load.

### Acknowledgements

S.C. has been associated with the Biotechnology Centre, Indian Institute of Technology, Kharagpur, for some years. Thanks are due to many of the members, and especially to Professor S. Dey, for some useful suggestions. J.C and S.C. thank Professor Bernard Prum of Laboratoire Statistique et Genome, La Genopole, for a discussion. S.C. is indebted to Professor Daniel Gautheret of TAGC INSERM ERM206, Marseilles, for his critical comments and suggestions.

### References

Antezana MA, Kreitman M. 1999. The non-random location of synonymous codons suggests that reading frame-independent forces have patterned codon preferences. *J Mol Evol* **49**: 36–43.

Ball LA. 1972. Implications of secondary structure in messenger RNA. *J Theor Biol* **36**: 313–320.

Ball LA. 1973. Secondary structure and coding potential of the coat protein gene of bacteriophage MS2. *Nature New Biol* **242**: 44–45.

Bell SJ, Forsdyke DR. 1999. Deviations from Chargaff's second parity rule correlate with direction of transcription. *J Theor Biol* **197**: 63–76.

Berg OG, Silva PJN. 1997. Codon bias in *Escherichia coli*: the influence of codon context on mutation and selection. *Nucleic Acids Res* **25**: 1397–1404.

Bernardi G, Bernardi G. 1986. Compositional constraints and genome evolution. *J Mol Evol* **24**: 1–11.

Brown JR, Doolittle WF. 1997. Archaea and the prokaryote-to-eukaryote transition. *Microbiol Mol Biol Rev* **61**: 456–502.

Bult CJ, White O, Olsen GJ, *et al.* 1996. Complete genome sequence of the methanogenic archaeon, *Methanococcus jannaschii*. *Science* **273**: 1058–1073.

Cavalier-Smith T. 2002. The neomuran origin of archaebacteria, the negibacterial root of the universal tree and bacterial megaclassification. *Int J Syst Evol Microbiol* **52**: 7–76.

Chargaff E. 1963. *Essays on Nucleic Acids*. Elsevier: Amsterdam.

Chattopadhyay S, Kanner WA, Chakrabarti J. 2002. DNA nucleotides: a case study of evolution. *Eur Phys J B* **26**: 393–398.

Dalgaard JZ, Garrett A. 1993. Archaeal hyperthermophilic genes. In *The Biochemistry of Archaea (Archaebacteria)*, Kates M, Kushner DJ, Matheson AT (eds). Elsevier: Amsterdam; 535–562.

Dang KD, Dutt PB, Forsdyke DR. 1998. Chargaff differences correlate with transcription direction in the bithorax complex of *Drosophila*. *Biochem Cell Biol* **76**: 129–137.

Fedorov A, Saxonov S, Gilbert W. 2002. Regularities of context-dependent codon bias in eukaryotic genes. *Nucleic Acids Res* **30**: 1192–1197.

Filipski J. 1990. Evolution of DNA sequences. Contributions of mutational bias and selection to the origin of chromosomal compartments. *Adv Mut Res* **2**: 1–54.

Forterre P, Elie C. 1993. Chromosome structure, DNA topoisomerases, and DNA polymerases in archaebacteria (archeae). In *The Biochemistry of Archaea (Archaebacteria)*, Kates M, Kushner DJ, Matheson AT (eds). Elsevier: Amsterdam; 325–345.

Friedman SM, Malik M, Drlica K. 1995. DNA supercoiling in a thermotolerant mutant of *Escherichia coli*. *Mol Gen Genet* **248**: 417–422.

Gaasterland T. 1999. Archaeal genomics. *Curr Opin Microbiol* **2**: 542–547.

Galtier N, Lobry JR. 1997. Relationships between genomic G + C content, RNA secondary structures, and optional growth temperature in prokaryotes. *J Mol Evol* **44**: 632–636.

Irwin B, Heck JD, Hatfield GW. 1995. Codon pair utilization biases influence translational elongation step times. *J Biol Chem* **270**: 22 801–22 806.

Karlin S, Mrázek J. 1996. What drives codon choices in human genes? *J Mol Biol* **262**: 459–472.

Kawarabayasi Y, Hino Y, Horikawa H, *et al.* 1999. Complete genome sequence of an aerobic hyper-thermophilic Crenarchaeon, *Aeropyrum pernix* K1. *DNA Res* **6**: 83–101.

Kawarabayasi Y, Hino Y, Horikawa H, *et al.* 2001. Complete genome sequence of an aerobic thermoacidophilic crenarchaeon, *Sulfolobus tokodaii* strain7. *DNA Res* **8**: 123–140.

Kawarabayasi Y, Sawada M, Horikawa H, *et al.* 1998. Complete sequence and gene organization of the genome of a hyper-thermophilic archaebacterium, *Pyrococcus horikoshii* OT3. *DNA Res* **5**: 147–155.

Kawashima T, Yamamoto Y, Aramaki H, *et al.* 1999. Determination of the complete genomic DNA sequence of *Thermoplasma volvanium* GSS1. *Proc Jpn Acad* **75**: 213–218.

Keeling PJ, Doolittle WF. 1995. Archaea: narrowing the gap between prokaryotes and eukaryotes. *Proc Natl Acad Sci USA* **92**: 5761–5764.

Klenk HP, Clayton RA, Tomb J, *et al.* 1997. The complete genome sequence of the hyperthermophilic, sulphate-reducing archaeon *Archaeoglobus fulgidus*. *Nature* **390**: 364–370.

Lao PJ, Forsdyke DR. 2000. Thermophilic bacteria strictly obey Szybalsky's transcription direction rule and politely purine-load RNAs with both adenine and guanine. *Genome Res* **10**: 228–236.

McVean GAT, Hurst GDD. 2000. Evolutionary liability of context-dependent codon-bias in bacteria. *J Mol Evol* **50**: 264–275.

Mrázek J, Kypr J. 1994. Biased distribution of adenine and thymine in gene nucleotide sequences. *J Mol Evol* **39**: 439–447.

Myllykallio H, Lopez P, Lopez-Garcia P, *et al.* 2000. Bacterial mode of replication with eukaryotic-like machinery in a hyperthermophilic archaeon. *Science* **288**: 2212–2215.

Ng WV, Kennedy SP, Mahairas GG, *et al.* 2000. From the cover: genome sequence of *Halobacterium* species NRC-1. *Proc Natl Acad Sci USA* **97**: 12 176–12 181.

Olsen GJ, Woese CR. 1993. Ribosomal RNA: a key to phylogeny. *FASEB J* **7**: 113–123.

Olsen GJ, Woese CR. 1997. Archaeal genomics: an overview. *Cell* **89**: 991–994.

Olsen GJ, Woese CR, Overbeek R. 1994. The winds of (evolutionary) change: breathing new life into microbiology. *J Bacteriol* **176**: 1–6.

Oshima T, Hamasaki N, Uzawa T, Friedman SM. 1990. Biochemical features of unusual polyamines found in the cells of extreme thermophiles. In *The Biology and Chemistry of Polyamines*,

Copyright © 2003 John Wiley & Sons, Ltd. 

Goldembeg SH, Algranati ID (eds). Oxford University Press: New York; 1–10.

Pühler G, Leffers H, Gropp F, *et al.* 1989. Archaebacterial DNA-dependent RNA polymerases testify to the evolution of the eukaryotic nuclear genome. *Proc Natl Acad Sci USA* **86**: 4569–4573.

Ruepp A, Graml W, Santos-Martinez ML, *et al.* 2000. The genome sequence of the thermoacidophilic scavenger *Thermoplasma acidophilum*. *Nature* **407**: 508–513.

Salser W. 1970. Discussion. *Cold Spring Harbor Symp Quant Biol* **35**: 19.

She Q, Singh RK, Confalonieri F, *et al.* 2001. The complete genome of the crenarchaeon *Sulfolobus solfataricus* P2. *Proc Natl Acad Sci USA* **98**: 7835–7840.

Smith DR, Doucette-Stamm LA, Deloughery C, *et al.* 1997. Complete genome sequence of *Methanobacterium thermoautotrophicum* ΔH: functional analysis and comparative genomics. *J Bacteriol* **179**: 7135–7155.

Sueoka N. 1995. Intrastrand parity rules of DNA base composition and usage biases of synonymous codons. *J Mol Evol* **40**: 318–325.

Sueoka N. 1999. Two aspects of DNA base composition: G + C content and translation-coupled deviation from intra-strand rule of A = T and C = G. *J Mol Evol* **49**: 49–62.

Szybalsky W, Kubinski H, Sheldrick P. 1966. Pyrimidine clusters on the transcribing strands of DNA and their possible role in the initiation of RNA synthesis. *Cold Spring Harbor Symp Quant Biol* **31**: 123–127.

Ussery DW. 2001. In *The Encyclopedia of Genetics*, Brenner S, Miller JH (eds). Academic Press: New York; 550–553.

Woese CR. 1987. Bacterial evolution. *Microbiol Rev* **51**: 221–271.

Woese CR, Fox GE. 1977. Phylogenetic structure of the prokaryotic domain: the primary kingdoms. *Proc Natl Acad Sci USA* **74**: 5088–5090.

Woese CR, Kandler O, Wheelis ML. 1990. Towards a natural system of organisms: proposal for the domains Archaea, Bacteria, and Eukarya. *Proc Natl Acad Sci USA* **87**: 4576–4579.

Zillig W, Palm P, Klenk H-P, *et al.* 1993. Transcription in Archaea. In *The Biochemistry of Archaea (Archaebacteria)*, Kates M, Kushner DJ, Matheson AT (eds). Elsevier: Amsterdam; 367–391.

Copyright © 2003 John Wiley & Sons, Ltd. 
